# Obstructive shock due to a spontaneous haemothorax caused by primary lung cancer

**DOI:** 10.1002/rcr2.1082

**Published:** 2023-01-02

**Authors:** Hiroe Aramaki, Kiyomitsu Fukaguchi, Hiroshi Yamagami, Yuki Maeda, Shun Tsunoda, Kenichiro Akazawa, Hiroshi Koyama

**Affiliations:** ^1^ Department of General Internal Medicine Shonan Kamakura General Hospital Kamakura Japan; ^2^ Division of Critical Care Shonan Kamakura General Hospital Kamakura Japan; ^3^ Department of Emergency Medicine Shonan Kamakura General Hospital Kamakura Japan; ^4^ Department of Haematology Shonan Kamakura General Hospital Kamakura Japan

**Keywords:** Haemothorax, lung neoplasms, shock, ultrasound imaging

## Abstract

We describe the case of a 67‐year‐old man with shock and hypoxemia. Chest X‐ray showed bilateral lung mass shadows and left pleural effusion with a mediastinal shift, suggesting malignancy. Physical examination and point‐of‐care ultrasound findings did not suggest obstructive or cardiac shock, but the patient had prolonged shock refractory to fluid and blood transfusion therapy. We inserted a drain into the left thoracic cavity, which enabled the patient to recover from shock. We diagnosed the patient with obstructive and hypovolemic shock due to spontaneous haemothorax caused by primary lung cancer. Tension haemothorax due to malignancy is rare, and when obstructive shock is combined with haemorrhagic shock, it can be very difficult to determine the cause of shock.

## Graphical abstract

We present a case of obstructive shock due to spontaneous haemothorax. Tension haemothorax due to malignancy is rare, and when obstructive shock is combined with haemorrhagic shock, it can be very difficult to determine the cause of shock.

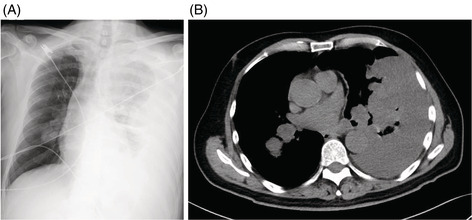

## INTRODUCTION

Haemothorax causes are classified as traumatic, iatrogenic, or spontaneous. Malignancy is rarely the cause of spontaneous haemothorax.^1^, ^2^ There are few reports of spontaneous haemothorax causing obstructive shock.^3^ We present a case of obstructive shock due to spontaneous haemothorax.

## CASE REPORT

A 67‐year‐old man without particular medical history presented to a clinic with a 2‐week history of gradually worsening dyspnoea. Chest X‐ray showed mass shadows in both lung fields, and an electrocardiogram revealed atrial fibrillation. Therefore, he was prescribed an anticoagulant (edoxaban 60 mg, once daily) and referred to a general hospital for further investigation. The following day, approximately 7–8 h after chest computed tomography (CT) was performed at the general hospital, his dyspnoea deteriorated rapidly. Finally, he called an ambulance and was admitted to our hospital.

On arrival, he was awake and afebrile, with a blood pressure of 123/85 mmHg; heart rate (HR), 151 bpm; respiratory rate, 38 breaths/min; and oxygen saturation, 99% under oxygen administration at 10 L/min with a non‐rebreather mask. On physical examination, decreased respiratory sounds in the left chest and cold extremities, but no jugular venous distention, were observed.

**FIGURE 1:**
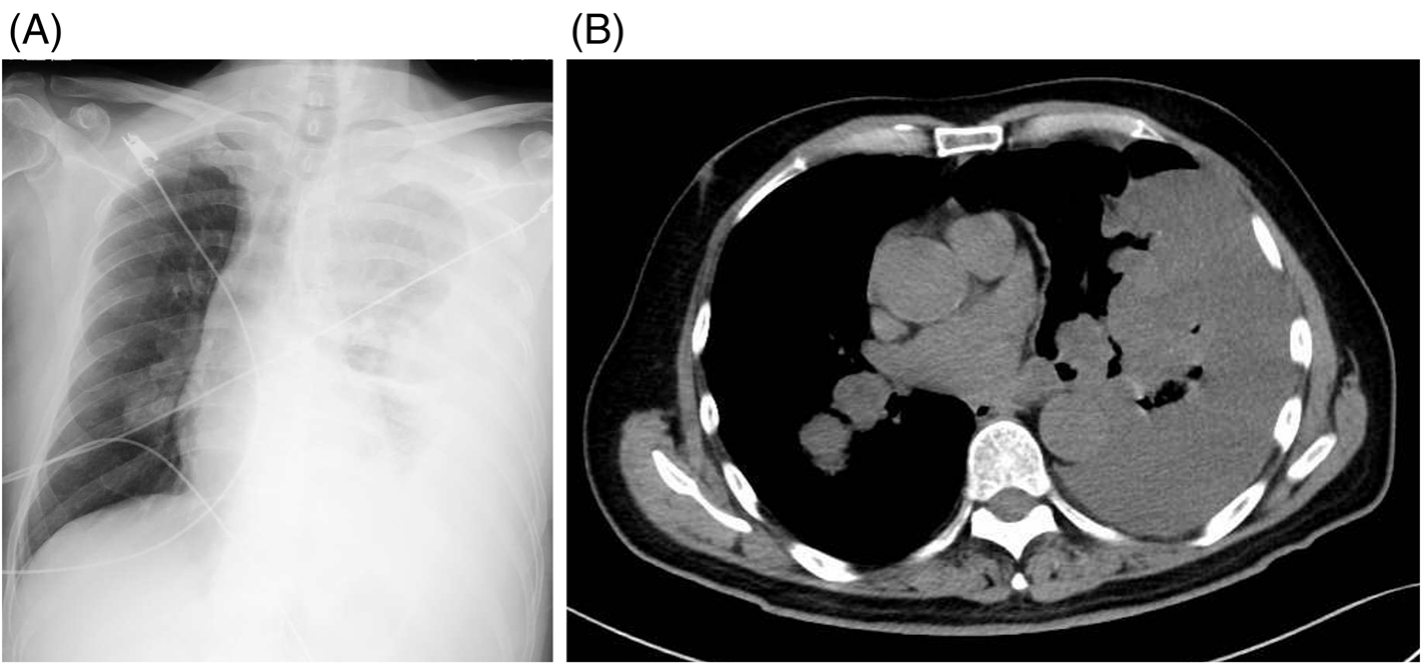
(A) Chest radiograph after emergency transport. (B) Plain CT image of the chest after emergency transport showing masses in both lung fields, left pleural effusion, and mediastinum deviated to the right

Arterial blood gas analysis showed the following: pH: 7.32, PaCO_2_: 20.2 mmHg, PaO_2_: 181.4 mmHg, and lactate: 6.96 mmol/L. Blood test results revealed the following: haemoglobin: 11.8 g/dL, prothrombin time (international normalized ratio): 2.13, and activated partial thromboplastin time: 30.5 s. Chest X‐ray revealed opacity of the left lung field with mild rightward deviation of the trachea. Plain CT showed left pleural effusion at 31.5 HU, rightward deviation of the mediastinum, and mass shadows in both lung fields (Figure 1). Pulmonary arteriography CT showed no evidence of thrombus. Emergency point‐of‐care ultrasound (POCUS) revealed an ejection fraction of approximately 50%, with no obvious pericardial effusion, wall asynergy, or valvular disease. Signs of right heart failure were not evident, with fair collapsibility of the inferior vena cava (IVC). Thoracocentesis with the removal of 100 ml bloody pleural effusion did not improve the haemodynamic condition.

We initially thought that shock and hypoxemia were caused by tumour‐related symptoms of chronic and continuous bleeding and obstructive atelectasis. Seven hours after admission, his condition deteriorated with haemodynamic instability, despite adequate fluid and blood transfusion, necessitating mechanical ventilation and administration of vasopressors. However, the shock was refractory to noradrenaline 0.33 μg/kg/min after 14 h of admission; therefore, we performed left chest tube thoracostomy considering the possibility of tension haemothorax. Approximately 500 ml of bloody pleural fluid was drained immediately after chest tube insertion, and the blood pressure markedly increased from 70/50 to 130/80 mmHg. We considered that the bloody pleural fluid increased the intrathoracic pressure and led to shock. Finally, we determined the main cause of shock to be an obstructive condition due to haemothorax. In total, 3000 ml of effusion was drained. Although contrast‐enhanced CT did not show extravasation from the tumour, we performed catheter embolisation for the inferior transverse and bronchial arteries to prevent re‐bleeding of the tumour on day 4. We also performed endobronchial ultrasound‐guided transbronchial needle aspiration on day 12. He was discharged on day 25. Biopsy revealed poorly differentiated adenocarcinoma; therefore, a diagnosis of primary lung adenocarcinoma cT4N3M1c Stage IV_b_ was made.

**FIGURE 2:**
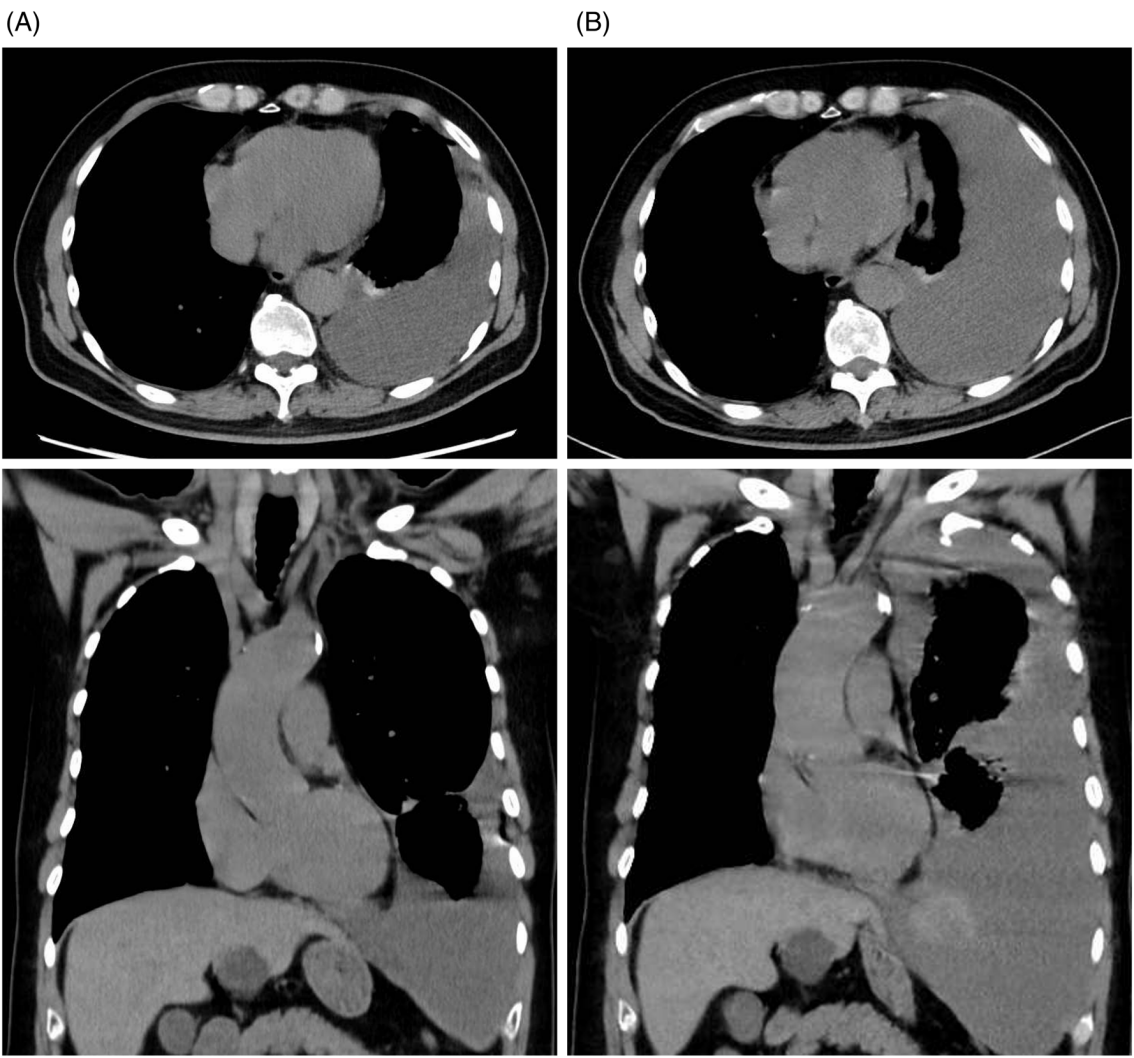
(A) Chest CT image taken by the previous physician at approximately 7–8 h before emergency transport. (B) Chest CT image taken after emergency transport. Increased pleural effusion and mediastinal deviation are observed

Comparing the CT images taken by the former physician retrospectively, we found an increase in left pleural effusion with the mediastinum oppressed to the right in approximately 7–8 h, indicating spontaneous bleeding (Figure 2).

## DISCUSSION

It took 14 h to recognize that the patient had haemothorax‐induced obstructive shock due to the difficulty in diagnosis.

Haemothorax is defined as a haematocrit value of pleural fluid of >50% blood.^1^ Pleural effusion is indistinguishable from blood based on appearance when the haematocrit value exceeds 5%; therefore, we cannot distinguish haematologic effusion from haemothorax. As bloody pleural effusions are commonly associated with lung cancer, it was difficult to consider bloody pleural effusion by thoracentesis as haemothorax, suggesting acute haemorrhage.

Most cases of haemothorax are traumatic or related to medical procedures, and spontaneous haemothorax is rare. Spontaneous haemothorax may be caused by malignancy, anticoagulants, ruptured blood vessels, endometriosis, pulmonary infarction, pneumothorax adhesions, and haematologic abnormalities such as haemophilia.^1^ Among malignant tumours, schwannomas of Von Recklinghausen disease and soft tissue tumours are common, while primary lung cancer is rare.^1^, ^4^ Although it is easy to anticipate that a traumatic haemothorax can rapidly increase and lead to shock, in this case, it was difficult to presume a rapid increase because the pleural effusion was thought to be related to a malignant tumour and gradually increasing. A previous study reported a case of spontaneous haemothorax caused by oral anticoagulants.^5^ Here, edoxaban prescribed the day before may have worsened bleeding, leading to shock.

To provide therapeutic intervention, we should identify the cause of shock. Some patients may have multiple factors, and physical examination and POCUS are useful in refining the diagnosis. In this case, there were no findings suggestive of obstructive shock from the initial treatment stage, such as IVC dilatation, right ventricular dilation, or pericardial effusion. Despite repeated evaluations before and after intubation or drainage, we had difficulty identifying signs of obstruction.

Tension haemothorax is caused by a decrease in venous return due to increased intrathoracic pressure, resulting in a decrease in preload and shock. This is similar to tension pneumothorax, although pneumothorax is caused by air and haemothorax is caused by blood. As tension haemothorax is associated with massive bleeding into the thoracic cavity, tension haemothorax is often associated with hypovolemic and obstructive features. The decreased blood volume, which is associated with haemothorax, made obstructive pathology difficult to recognize.

In conclusion, the diagnosis of acutely worsened spontaneous haemothorax is difficult by physical examination or POCUS because of its combination of hypovolemic and obstructive features.

## AUTHOR CONTRIBUTIONS

Hiroe Aramaki is the corresponding author and principal author. Kiyomitsu Fukaguchi, Hiroshi Yamagami, and Hiroshi Koyama substantially made contributions to the conception of the work and contributed to the revision of the manuscript drafts. Yuki Maeda, Shun Tsunoda, and Kenichiro Akazawa offered helpful advice. All authors have approved the submitted version of the manuscript and agreed to be accountable for any part of the work.

## CONFLICT OF INTEREST

None declared.

## ETHICS STATEMENT

The authors declare that appropriate written informed consent was obtained for the publication of this manuscript and accompanying images.

## Data Availability

Data sharing is not applicable to this article as no new data were created or analyzed in this study.

## References

[1] Azfar Ali H , Lippmann M , Mundathaje U , Khaleeq G . Spontaneous hemothorax: a comprehensive review. Chest. 2008;134:1056–65. 10.1378/chest.08-0725 18988781

[2] Chou SH , Cheng YJ , Kao EL , Chai CY . Spontaneous haemothorax: an unusual presentation of primary lung cancer. Thorax. 1993;48:1185–6. 10.1136/thx.48.11.1185 8296269 PMC464922

[3] Föhrding LZ , Sellmann T , Angenendt S , Kindgen‐Milles D , Topp SA , Korbmacher B , et al. A case of lethal spontaneous massive hemothorax in a patient with neurofibromatosis 1. J Cardiothorac Surg. 2014;9:172. 10.1186/s13019-014-0172-y 25348553 PMC4223165

[4] Patrini D , Panagiotopoulos N , Pararajasingham J , Gvinianidze L , Iqbal Y , Lawrence DR . Etiology and management of spontaneous haemothorax. J Thorac Dis. 2015;7:520–6. 10.3978/j.issn.2072-1439.2014.12.50 25922734 PMC4387396

[5] Yan DT , Heng RGK , Ng HJ . Massive spontaneous haemothorax after rivaroxaban therapy for acute pulmonary embolism. Eur J Case Rep Intern Med. 2019;6:1236. 10.12890/2019_001236 PMC6822669 31742199

